# Bayesian Modeling of Perceived Surface Slant from Actively-Generated and Passively-Observed Optic Flow

**DOI:** 10.1371/journal.pone.0018731

**Published:** 2011-04-14

**Authors:** Corrado Caudek, Carlo Fantoni, Fulvio Domini

**Affiliations:** 1 Department of Psychology, Università degli Studi di Firenze, Firenze, Italy; 2 Center for Neurosciences and Cognitive Systems, Istituto Italiano di Tecnologia, Rovereto, Italy; 3 Department of Cognitive, Linguistic and Psychological Sciences, Brown University, Providence, Rhode Island, United States of America; University of Leuven, Belgium

## Abstract

We measured perceived depth from the optic flow (a) when showing a stationary physical or virtual object to observers who moved their head at a normal or slower speed, and (b) when simulating the same optic flow on a computer and presenting it to stationary observers. Our results show that perceived surface slant is systematically distorted, for both the active and the passive viewing of physical or virtual surfaces. These distortions are modulated by head translation speed, with perceived slant increasing directly with the local velocity gradient of the optic flow. This empirical result allows us to determine the relative merits of two alternative approaches aimed at explaining perceived surface slant in active vision: an “inverse optics” model that takes head motion information into account, and a probabilistic model that ignores extra-retinal signals. We compare these two approaches within the framework of the Bayesian theory. The “inverse optics” Bayesian model produces veridical slant estimates if the optic flow and the head translation velocity are measured with no error; because of the influence of a “prior” for flatness, the slant estimates become systematically biased as the measurement errors increase. The Bayesian model, which ignores the observer's motion, always produces distorted estimates of surface slant. Interestingly, the predictions of this second model, not those of the first one, are consistent with our empirical findings. The present results suggest that (a) in active vision perceived surface slant may be the product of probabilistic processes which do not guarantee the correct solution, and (b) extra-retinal signals may be mainly used for a better measurement of retinal information.

## Introduction

The current models of active Structure-from-Motion (SfM) are based on the Helmholtzian account of perception as inverse inference [1]–[5]. According to this approach, the goal of the perceptual system is to infer from the sensory evidence the environmental three-dimensional (3D) shape most likely to be responsible for producing the sensory experience. In order to obtain this goal, the current approach inverts the generative model for the optic flow. Mathematically, this corresponds to an application of Bayes' rule in which first-order optic flow information is combined with information about the observer's motion provided by proprioceptive and vestibular signals [6]. The solution of this “inverse-optics” problem can produce the correct result if some assumptions about the distal objects are satisfied and if the extra-retinal signals are measured with high precision [3].

**Figure 1:**
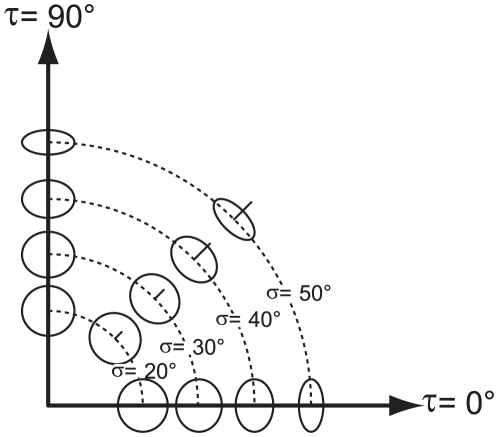
The two degrees of freedom of surface orientation. A set of circular patches is used to illustrate the slant (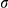) and tilt (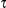) components of surface orientation [40]. The line at the center of each patch is aligned in the direction of the surface normal. The slant 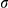 is defined by the tangent of the angle between the normal to the surface and the line of sight 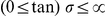. The tilt 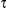 is defined as the angle between the 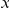-axis of the image plane and the projection into the image plane of the normal to the surface (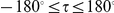). The dotted lines identify patches with same slant but different tilt magnitudes.

An alternative theory hypothesizes that the visual system estimates the metric properties of local surface orientation by using retinal information alone. Retinal information “directly” specifies the 3D affine properties of the distal object (such as the parallelism of local surface patches or the relative-distance intervals in parallel directions), but it does not allow a unique determination of its Euclidean metric properties, such as slant [7] (see Figure 1). [8] proposed that the perception of local surface slant can be understood in terms of a probabilistic estimation process. Consider a property 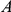 of the optic flow which is related to the distal property 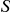 through a one-to-many mapping. Any estimate 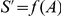 based solely on 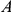 will produce an error equal to 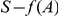. Through learning, however, the visual system may select the function 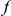 that minimizes 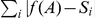, where 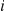 indexes the instances 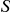 that could have produced 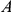. This approach has proven adequate to explain human passive SfM, but it could be applied to active SfM as well.

A fundamental difference between these two approaches is that only the first one makes use of information about ego-motion. This difference is very important. In fact, empirical results show that perceived surface tilt depends critically on ego-motion information. A particularly convincing demonstration in this respect has been provided by [9]. In the “active” condition, the observer translated along the 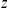-axis while fixating a planar surface with 90 tilt and undergoing rotation about the horizontal axis. The rotation of the surface was paired with the observer's motion, so as to generate a pure compression optic flow. In the “passive” condition, the same optic flow was “replayed” to a stationary observer. Tilt perception was veridical when ego-motion information was available (perceived tilt was 90), but not for the passive observer (perceived tilt was either 0 or 180) – see also [10]–[13].

The purpose of the present investigation is to determine whether observers use information about the speed of head motion to estimate surface slant. To this purpose, we compared the judgments of local surface slant provided by active and passive observers to the estimates provided by two Bayesian models. The two models were constructed (a) by taking into account information about the observer head motion, and (b) without taking into account information about the observer head motion. The empirical data were obtained by asking observers to judge the local slant of virtual and physical planar surfaces from the optic flows generated by normal or slower head translation velocities.

### Surface slant and first-order optic flow

**Figure 2:**
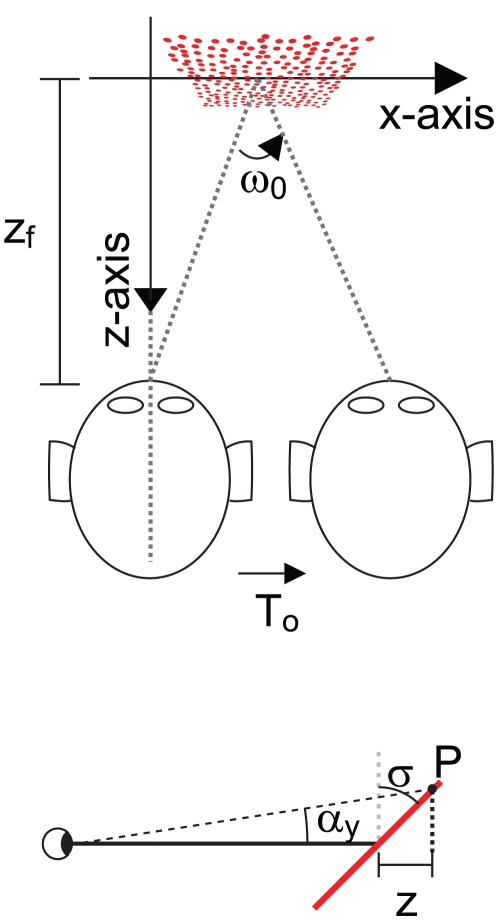
Schematic of stimulus geometry. Top panel: bird-view of the viewing geometry. The 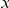 and the 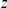 axes represent the horizontal image dimension and the line of sight, respectively; 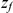 is the viewing distance, 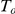 is the horizontal head translation velocity, and 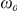 is the relative angular velocity produced by the motion of the observer's head. Bottom panel: side view of the viewing geometry. 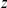 represents the relative depth of the point **P** with respect to the fixation point; 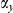 represents the elevation of the point **P** with respect to the optical axis; 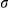 is the tangent of the angle between the surface (represented by the red segment) and the fronto-parallel plane.

Consider a coordinate system centered at the observer's viewpoint, with the 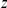 axis orthogonal to the observer's frontal-parallel plane (see Figure 2). Suppose that the observer fixates the surface's point located at 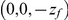, where 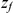 is the viewing distance. If the observer translates in a direction orthogonal to the line of sight, with translational velocity 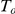, or the surface rotates with angular velocity 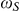, then the texture elements on the surface will project onto the image plane a velocity field which can be locally described by the following equation:
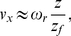
(1)where 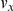 is the retinal angular velocity, 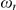 is the angular velocity resulting from the relative rotation between the observer and the surface, and 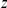 is the relative depth of each surface point with respect to the fixation point.

In the present investigation, we only consider planar surfaces slanted by an angle 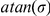 along the vertical (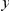) dimension. Such surfaces are defined by equation
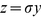
(2)which, substituted in Eq. 1, gives:
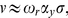
(3)where 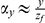 is the vertical elevation of a generic feature point. The deformation (*def*) component (*i.e.*, the gradient) of the velocity field – which is zero along the horizontal dimension for our stimuli – is given by
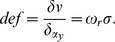
(4)Eq. 4 is a good approximation of the local velocity field produced by a surface patch subtending up to 8 of visual angle. Importantly, Eq. 4 reveals that the gradient of the velocity field is *not* sufficient to specify the slant of the surface. In order to specify 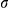, in fact, the knowledge of the relative rotation 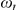 between the observer and the planar surface is required. Note that, in general, 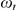 depends both on the surface's rotation about the vertical axis (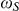) and on the translation of the observer:
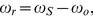
(5)where 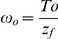 denotes the relative velocity of the surface resulting from the movement of the observer in an egocentric reference frame.

In general, the ambiguity of *def* could be solved if the visual system were able to accurately measure the second-order optic flow (*i.e.*, the image accelerations), but several studies show that this is not the case [14]–[18]. Alternatively, *def* can be disambiguated by combining the information provided by the first-order optic flow and the extra-retinal signals, if some assumptions are met (see next Section).

### Bayesian slant estimation from retinal, vestibular, and proprioceptive information

The ambiguity of *def* can be overcome by the active observer under the assumption that the object is stationary – a reasonable assumption in many real-world situations [12]. If the object is stationary, 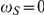 and the relative rotation between the observer and the surface is equal-and-opposite to the observer's motion: 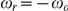. If information about 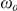 is obtained from proprioceptive and vestibular signals, it is thus possible to estimate 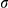.

The Bayesian model presented by Colas and collaborators formalizes this idea [6]. The uncertainty in the estimation of the relative motion  is described by a Gaussian distribution 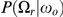 centered at 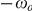 and having an arbitrary standard deviation 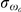 (here and in the following we use capital letters to indicate random variables). The spread of this Gaussian distribution encodes the noise in the measurement of the vestibular and proprioceptive signals and the possibility that the surface undergoes a rotation independent from the observer's motion. By centering this probability distribution at 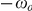, Colas et al. implement the *stationarity assumption*, that is, they favor the solutions in which the optic flow is produced by the observer's motion [12]. Colas et al. also consider the possibility that the optic flow is not measured accurately, or is produced by some degree of non-rigid motion. Under these circumstances, the surface slant 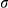 combined with the relative motion 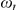 does not produce a unique *def* value. This further source of uncertainty is described by a Gaussian distribution 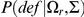 centered at 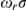 with an arbitrary standard deviation 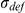. By centering this probability distribution at 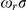, Colas et al. implement the *rigidity assumption*, that is, they favor the solutions in which *def* is produced by a rigid rotation. A further assumption is that the slant of the surface does not depend on the relative motion between the surface and the observer.

Under these assumptions, the problem of estimating local surface slant given the knowledge of *def* and 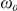 (the observer's motion) becomes the problem of identifying the density function 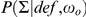. This probability density function can be found through Bayes' theorem by applying the rules of marginalization and probability decomposition.

**Figure 3:**
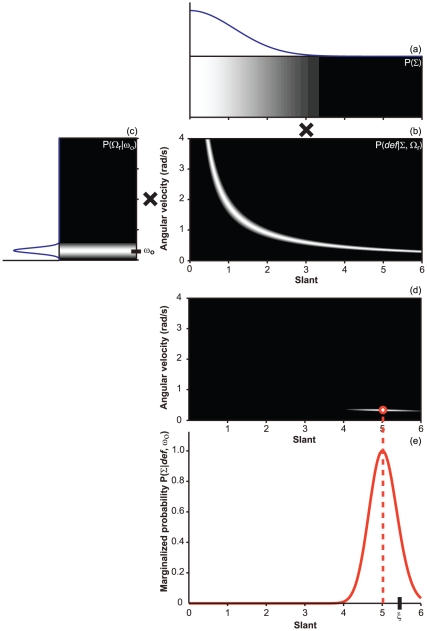
Recovery of surface slant according to **Eq. 14**. Intensity corresponds to probability. The values reported in the plot refer to the case of a static plane slanted by 80 (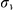) around the horizontal axis and viewed by an active observer performing a lateral head translation at a speed that produces a relative angular-rotation velocity of 0.32 rad/s (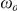). **Panels a - e**: method for calculating the posterior distribution. **a**. Prior for frontal-parallel 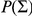 modeled as a (half) Gaussian distribution centered at zero. **b**. Likelihood function 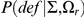 generated by assuming that the *def* measurements are corrupted by Gaussian noise. **c**. Uncertainty of the relative rotation between the observer and the planar surface 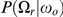 modeled as a Gaussian distribution centered on the true value 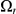. **d**. Product of the likelihood, the prior for 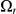, and the prior for 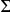. **e**. Posterior distribution produced by the marginalization over 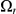. The median of the posterior distribution (dotted line) gives the optimal estimate of surface slant based on the knowledge of *def* and 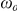. The model's prediction (the value 5 in the figure) gets more and more close to the “true” value of the slant (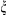) as 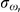 decreases.

From the definition of the conditional probability 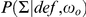, by marginalizing over 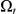, we obtain
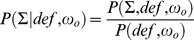
(6)

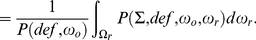
(7)By the chain rule, we can write
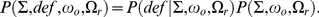
(8)Moreover,
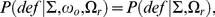
(9)because, under the rigidity assumption, *def* depends only on the distal slant 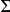 and the relative rotation 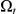;
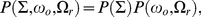
(10)because surface slant is independent from the observer's relative motion and from the egocentric motion;
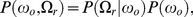
(11)because of the chain rule. Therefore, 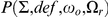 can be re-written as
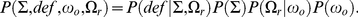
(12)By virtue of Eq. 12, Eq. 7 takes the form
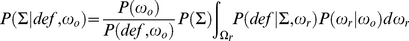
(13)

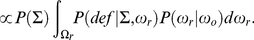
(14)In conclusion, Eq. 14 provides a possible solution to the “inverse optics” problem of estimating local surface slant from the deformation of the optic flow (see Figure 3). If 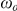 and *def* are measured with no error, then 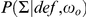 peaks at the true slant value (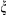) when the distal surface is stationary. In the presence of measurement errors, instead, the estimated slant will be biased. The magnitude of this bias depends on the precision with which 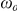 (the observer's motion) and *def* are measured: the larger 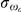, the larger the under-estimation of slant.

### Bayesian slant estimation from retinal information alone

**Figure 4:**
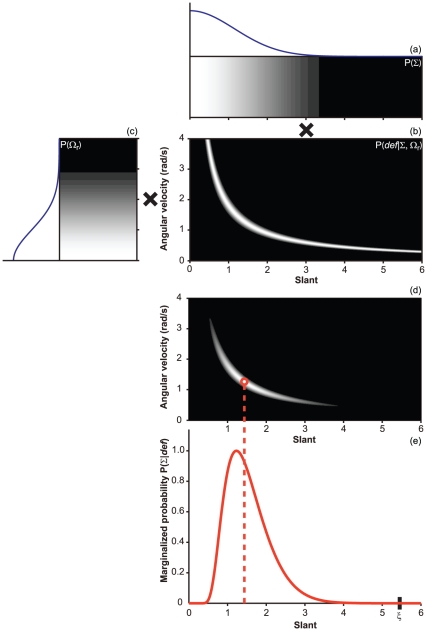
Recovery of surface slant according to **Eq. 15**. The values reported in the figure refer to the stimulus conditions used in Figure 3. Differently from the “inverse-optics” approach, in this case the distribution 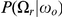 is non-informative. Note that, after computing the product of the likelihood, the prior for 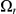, and the prior for 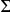, the marginalization over 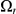 produces a posterior distribution that is very different from what is shown in Figure 3. The estimate of surface slant is given by the median of the posterior distribution (dotted line). The posterior median corresponds the point on the hyperbola closest to the origin of the Cartesian axes. The Bayesian posterior median estimator (which is equal to 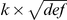) is represented by the red dot in panel 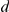.

We propose that the visual system estimates surface slant without considering the information about head translation velocity (see Figure 4). With reference to the Bayesian model discussed in the previous section, this means that 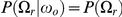, where 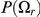 is the *a priori* distribution of a random variable representing the amount of relative rotation between the observer and the surface. In this case, Eq. 14 reduces to
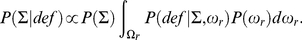
(15)


Domini and Caudek showed that this account is sufficient for predicting perceived slant from the optic flow in the case of the passive observer [19]–[28]. They showed that the center of mass of the distribution described by Eq. 15 is equal to 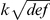, with 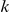 depending on the spreads of the prior distributions of 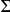 and of 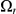 [8]. The center of mass as an estimate of 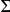 is equivalent to the posterior median, which is the Bayes estimator for the absolute error loss. Indeed, it has been shown that Eq. 15 is a particular case of Eq. 14: The two accounts are indistinguishable when information about the head's translation is unavailable, like in the case of the passive observer [6]. Eqs. 14 and 15, instead, make different predictions for the active observer, when head translation velocity is manipulated.

The importance of 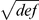 for the perceptual recovery of local surface slant from the optic flow has been highlighted by [8], [24]. *def* is a one-parameter family of 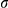 (surface slant) and 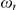 (relative angular rotation) pairs, but not all possible 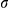, 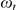 pairs are equally likely. Under the assumption of uniform prior distributions (bounded between 0 and 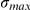, and between 0 and 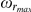) for 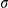 and 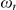, the conditional probability of a 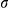, 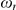 pair given *def* is not uniform, but it has a maximum equal to 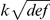, with 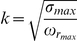 (see [24]).

### Rationale of the Experiments

Eqs. 14 and 15 provide two alternative models for the perceptual derivation of surface slant from the optic flow in active vision. The purpose of the present investigation is to contrast them by comparing their predictions to the behavioral data obtained when head translation velocity is manipulated.

In the present experiments, observers were required to produce two different head translation velocities. The first was comparable to the peak horizontal head velocity during normal locomotion [29], the second was 80% slower. This experimental manipulation (a) does not affect the estimate of local surface slant according to Eq. 14 (by assuming that the measurement noise of 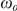 remains unaltered), and (b) can strongly affect the estimate of local surface slant according to Eq. 15 (because head translation velocity is proportional to 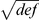).

## Results

### Perceived surface slant

**Figure 5:**
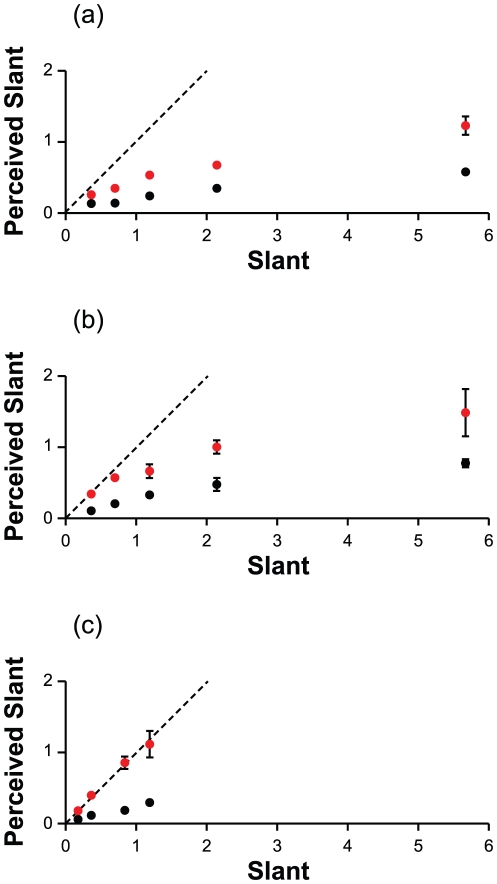
Perceived slant as a function of simulated slant. Fast and slow head translation velocities are coded by red and black, respectively. The values are expressed in terms of the tangent. The dashed lines indicate veridical performance. Vertical bars represent 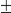 1 standard error of the mean. (a) *Passive-viewing with a virtual surface*. The interaction Slant 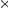 Velocity is significant, 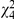 = 51.04, 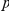 = .001. The marginal effect of the head's translation velocity is significant, 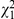 = 7.48, 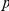 = .006: On average, the amount of reported slant is 51% lower for the slow than for the fast head's translation velocity. (b) *Active-viewing with a virtual surface*. The interaction Slant 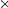 Velocity is significant, 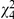 = 47.31, 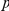 = .001: The effect of simulated slant on the response is larger for the faster head's translation velocity. The marginal effect of head's translation velocity is significant, 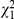 = 5.62, 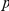 = .018: On average, the amount of reported slant is 60% lower for the slow than for the fast head's translation velocity. (c) *Active-viewing with a physical surface*. The interaction Slant 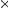 Velocity is significant, 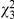 = 40.81, 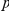 = .001. The marginal effect of head's translation velocity is significant, 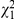 = 12.96, 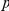 = .001: On average, the amount of reported slant is 75% lower for the slow than for the fast head's translation velocity.

Active and passive observers judged the perceived slants of virtual or physical planar surfaces. The results indicate that the judgements made by the observers are systematically biased by the head translation velocity (see Figure 5). The same qualitative trends are found for the active and passive viewing of a virtual surface, and for the active viewing of a physical surface.

**Figure 6:**
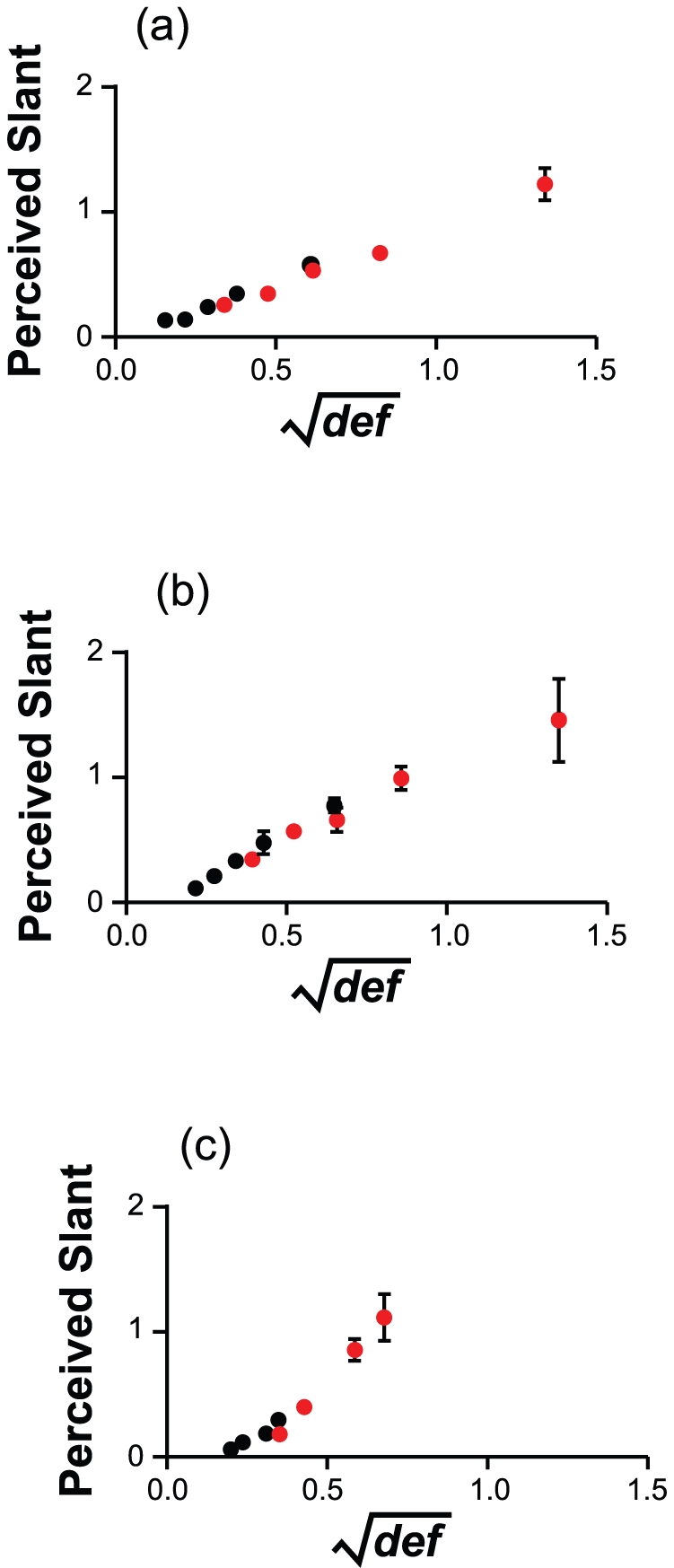
Perceived slant as a function of 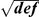. Fast and slow head translation velocities are coded by red and black, respectively. The values are expressed in terms of the tangent. Vertical bars represent 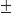 1 standard error of the mean. (a) *Passive-viewing with a virtual surface*. There is no significant interaction between 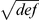 and head translation Velocity, 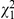 = 0.43, 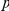 = .513. There is no significant effect of head translation Velocity, 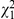 = 0.57, 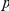 = .449. In a no-intercept model with 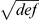 as the only predictor, the slope is 1.04, 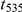 = 20.95, 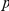 = .001, and 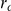 = .74. No improvement of fit is found if simulated slant is added to such model, 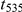 = 0.455. For a baseline model with only the individual differences, 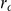 is equal to .45. (b) *Active-viewing with a virtual surface*. There is no significant interaction between 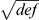 and head translation Velocity, 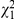 = 2.50, 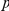 = .114; there is not significant effect of head translation Velocity, 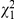 = 0.25, 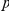 = .620. In a no-intercept model with 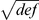 as the only predictor, the slope is 1.88, 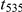 = 16.46, 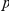 = .001, and 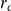 = .69. No improvement of fit is found if simulated Slant is added to such model, 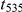 = −0.22. For a baseline model with only the individual differences, 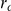 is equal to .33. When analyzing together the data of the passive and active viewing of the virtual surfaces, significant effects are found for 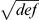, 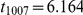, 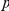 = .001, and condition (indicating a smaller response for the passive observer), 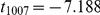, 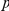 = .001. For this model, 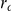 is equal to .90. When controlling for 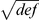, the effect of simulated Slant is not significant, 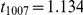. For a baseline model with only the individual differences, 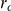 is equal to .43. The estimated standard deviation of the residuals is equal to 0.218 on the scale of the tangent of the slant angle. Given that mean perceived slant is 0.493, the coefficient of variation is equal to 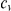 = 0.44. (c) *Active-viewing with a physical surface*. For a no-intercept model with the square root of *def* as the only predictor, 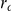 = .72. For a baseline model with only the individual differences, 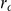 = .44. If head translation velocity is added to the model including *def* as predictor, 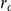 increases to .74; 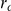 increases to .75 if the interaction between the two predictors is allowed. Even though this increase in the model's fit is statistically significant, 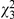 = 30.15, 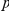 = .001, the effect size (as measured by 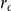) is very small. No improvement of fit is found when adding the simulated Slant predictor, 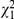 = 0.42, 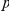 = .518. In the simpler (no-intercept) model with the 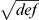 predictor, the slope is 2.41, 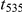 = 12.29, 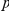 = .001.

According to the Bayesian model described in Eq. 15, perceived surface slant depends only on the square root of *def*. For the active and passive viewing of virtual planar surfaces, the observers' judgments of slant complied with this prediction (see Figure 6). For the active viewing of physical surfaces, 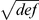 was not the only determinant of the perceptual response, but the additional contribution of the head translation velocity was negligible. In the present investigation, therefore, there is no evidence that simulated slant contributes to the perceptual response beyond what 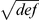 can explain.

### Implementation of the Bayesian models

#### Estimation of surface slant by taking into account head translation velocity

**Figure 7:**
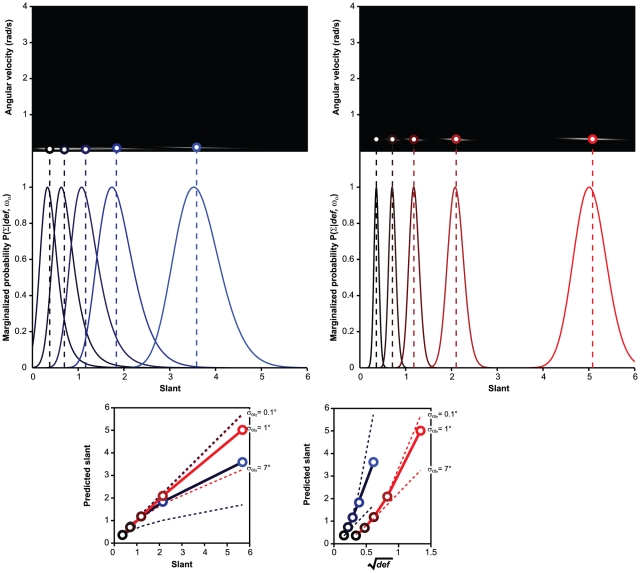
Monte Carlo simulation for the model of **Eq. 14**. **Top panels**: posterior distributions 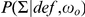 for five simulated slant magnitudes. Intensity corresponds to probability. The posterior distributions are computed by setting the mean of the Gaussian distribution 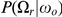 to the values of 0.32 or 0.07 rad/s. These values correspond to the empirical average of, respectively, the normal (right) or slow (left) head translation velocity in our experiment. **Middle panels**: marginalization of the posterior distributions over 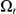. The simulated slant magnitudes are coded by color, ranging from black (20) to saturated blue/red (80). The dotted lines identify the medians of the marginalized posterior distributions. **Bottom panels**. Optimal estimation for surface slant plotted as a function of the simulated slant magnitudes (left) and 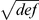 (right). Full lines: results obtained by setting 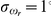; dashed lines: results obtained by setting 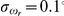 or 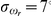. Blue and red colors code the slow and fast head translation velocities, respectively.

Figure 7 illustrates the process of slant estimation according to Eq. 14: The Bayesian estimator is plotted as a function of simulated slant (bottom left panel) and as a function of 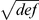 (bottom right panel). When the head translation velocity 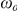 is measured accurately, the Bayesian estimator is veridical; as the uncertainty about 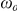 increases, the Bayesian estimator becomes more and more biased. The slant estimates expressed as a function of 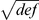 lie on two different curves, regardless of the size of 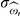 (bottom right panel). The offset between these two curves depends on the amount of measurement error and on the mean of the distribution 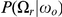, which depends on the head translation velocity.

#### Estimation of surface slant from retinal information alone

**Figure 8:**
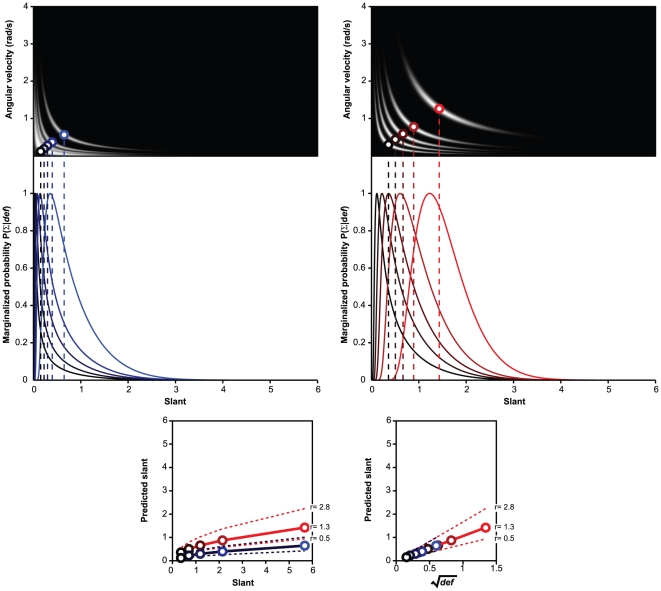
Monte Carlo simulation for the model of **Eq. 15**. The Monte Carlo simulation was carried out by setting the stimulus parameters to the same values as in Figure 7. **Top panels**: posterior distributions 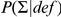. The outputs of the simulations for the slow and fast head translation velocities are shown on the left and on the right, respectively. **Middle panels**: marginalization of the posterior distributions over 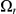. The dotted lines identify the medians of the marginalized posterior distributions. **Bottom panel**: estimates of surface slant plotted as a function of simulated slant (left) and as a function of 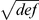 (right). Blue and red colors code the slow and fast head translation velocities, respectively. The parameter 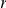 represents the square root of the ratio between the standard deviations of the prior distributions for 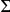 and 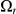.

Figure 8 illustrates the process of slant estimation according to Eq. 15: The Bayesian estimator is plotted as a function of simulated slant (bottom left panel) and as a function of 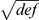 (bottom right panel). When plotted as a function of 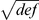, the slant estimates for the two translation velocities lie on the same straight line. The parameter 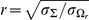 is the slope of the linear relation between perceived slant and 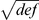.

#### Perceived surface slant and Bayesian modeling

**Figure 9:**
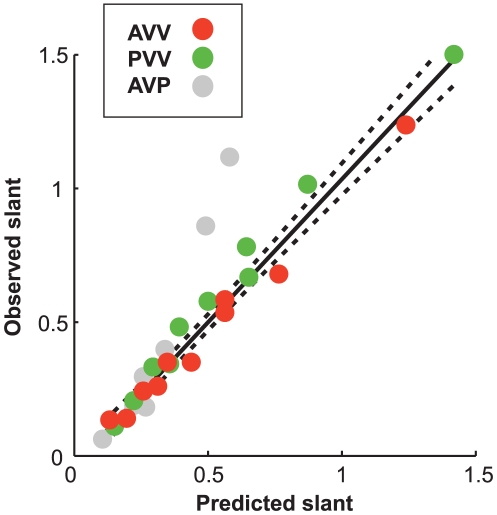
Perceived slant as a function of the predictions of **Eq. 15**. Points of the same color represent different simulated Slant magnitudes. The predicted values are obtained by setting 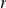 (the square root of the ratio between the standard deviations of the prior distributions for 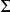 and 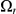) equal to the slope of the linear relation between perceived slant and 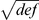 in each viewing condition. AVV: Active Viewing of a Virtual surface; PVV: Passive Vision of a Virtual surface; AVP: Active Viewing of a Physical surface. For the data in the figure, the least-squares regression line has an intercept of −0.03, 95% *C.I.* [−0.09, 0.02], and a slope of 1.07, 95% *C.I.* [0.97, 1.16]; 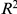 = .95.

Eq. 15 offers a clear advantage over Eq. 14 in predicting the observers' responses (see Figure 9). If the uncertainty about 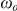 is not negligible, the Bayesian estimates of Eq. 14 expressed as a function of 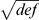 lie on two separate curves and are unable to reproduce the qualitative trends in the experimental data (see Figures 5, 6, and 7, 8). This lack of fit can be contrasted with the excellent correspondence between the slant estimates of Eq. 15 and the observers' judgments.

## Discussion

Under some assumptions, the optic flow can be used, together with other signals, to infer both the ordinal properties (*e.g.*, tilt) and the Euclidean metric properties (*e.g.*, slant) of the visual scene. By using sophisticated head-tracking techniques with high spatiotemporal resolution, we manipulated the information content of the stimuli to generate optic flows corresponding to (a) the active viewing of a virtual surface, (b) the passive viewing of a virtual surface, and (c) the active viewing of a physical surface. We also varied the head translation velocity (normal or slower). The observers' judgments of perceived surface slant were then compared to the Bayesian estimates computed with and without taking into account the translational velocity of the head (Eqs. 14 and 15, Figures 7 and 8).

The observers' responses are markedly different from the Bayesian estimates derived by combining optic flow and head velocity information (Eq. 14, Figures 5 and 6). The empirical data from the active and passive viewing of virtual planar surfaces, conversely, are consistent with the Bayesian estimates computed without considering head velocity information (Eq. 15, Figures 9).

For the slant judgments of physical planar surfaces, the Bayesian model of Eq. 15 explains a large amount of the variance, but a very small portion of additional variance is accounted for by the head translation velocity (Figure 6, bottom panel). The Bayesian model of Eq. 14, which takes head velocity into account, fits the data much worse. In the present research, this effect is small but warrants further research. In a follow-up experiment (not described here), we found that the monocular cues provided by our physical stimuli were not sufficient for an immobile observer to successfully discriminate between two surfaces slanted +45  or −45  (surface tilt was constant). Together with the findings of our main experiment, these results suggest that, although uninformative by themselves, monocular cues can produce some form of “enhancement” of the perceptual response, when they are presented together with the optic flow and with vestibular and proprioceptive information [30].

The slope of the linear relation between perceived surface slant and 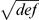 varies across the three viewing conditions: it is shallower for the passive viewing of a virtual planar surface, it increases for the active viewing of a virtual surface, and it is the largest for the active viewing of a physical surface. We may expect a different visual performance for passive and active SfM, and for virtual and physical stimuli. The present results suggest, however, that more complete stimulus information does not necessarily result in better (more veridical) performance: *A stronger effect of def does not guarantee* a more accurate response. Perceived slant is strongly affected by *def* despite the fact that there is no a “one-to-one” correspondence between *def* and distal surface slant.

Animal studies [31] and human experiments [32], [33] identify MT (MT+ in humans) as the brain area involved in SfM processing. It has also been shown that MST integrates MT inputs with vestibular signals originating from a different (currently unidentified) neural pathway [34], [35]. The integration of visual and vestibular information in MSTd is consistent with both the Bayesian models discussed here (Eqs. 14 and 15). Such integration could mean that (a) the visual system uses extra-retinal signals to discount head motion from the optic flow in order to encode a world-centered representation of the 3D objects [11], or (b) non-visual information about self-motion is used as a retinal stabilization factor for a better measurement of the optic flow [36], [37]. The present behavioral results, however, favor this second hypothesis.

In conclusion, the present data and simulations do not indicate so much that, by disregarding vestibular and proprioceptive information, the visual system uses a suboptimal strategy for estimating surface slant from the self-generated optic flow. Instead, they suggest that, even though it does not always guarantee a veridical solution to the SfM problem, the mapping between the deformation component of the optic flow and the perceived surface slant may be the most efficient choice for a biological system [38], [39]. An issue that remains to be investigated is whether and how learning provides effective visual and haptic feedback for scaling *def* information.

## Methods

### Ethics Statement

Experiments were undertaken with the understanding and written consent of each subject, with the approval of the Comitato Etico per la Sperimentazione con l'Essere Umano of the University of Trento, and in compliance with national legislation and the Code of Ethical Principles for Medical Research Involving Human Subjects of the World Medical Association (Declaration of Helsinki).

### Participants

Thirty-four undergraduate students at the University of Parma, Italy, participated in this experiment. All participants were naïve to the purposes of the experiment and had normal or corrected-to-normal vision.

### Apparatus

The orientation of the participant's head and the translational head displacements were recorded by an Optotrak 3020 Certus system. Two sensors recovered the 3D position data of two infrared emitting diodes (markers on an eyeglass frame) aligned with the observer inter-ocular axis. The signals emitted by the markers were used to calculate the 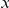, 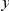, 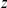 coordinates of the observers' viewpoints in order to update the geometrical projection of a random-dot planar surface in real time. Displays were monocularly viewed through a high-quality front-silvered mirror (150 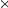 150 mm) placed at eye-height in front of the observer's central viewing position and slanted 45  away from the monitor and the observer's inter-ocular axis. The effective distance from the pupil to the center of the screen was 860 mm. Only the central portion of the surface was left visible to the observer through a black mask with an irregularly-shaped central aperture (about 70 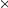 70 mm) superimposed on the screen. A chin-rest was used to prevent head movements in the passive-vision condition.

A custom Visual C++ program supported by OpenGL libraries and Optotrak API routines was used for stimulus presentation and response recording. The same program also controlled the orientation of a physical planar surface that, in a separate block of trials, was placed at a distance of 760 mm in front of the observer. The boundary of the physical surface was occluded by the same mask used for the virtual displays. This aperture was closed when the surface's orientation was changed.

### Stimuli

The simulated displays were random arrangements of (1 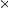 1 mm) antialiased red dots simulating the projection of a static planar surface centered on the image screen and with a variable slant about the horizontal axis (virtual planar surfaces: 20, 35, 50, 65, and 80; physical planar surfaces: 10, 20, 40, and 50). The surface tilt was constant (90). About 100 dots were visible through the irregular aperture occluding the outer part of the screen. To remove texture (non-motion) cues, the dots were randomly distributed into the projected image (not on the simulated surface). On each frame of the stimulus sequence, the 2D arrangement of the dots was varied depending on the observer's head position and orientation with respect to the simulated surface. The dots on the simulated planar surface were projected onto the image plane (CRT screen) by using a generalized perspective pinhole model with the observer's right eye position as the center of projections. The position of the observer's right eye was sampled at the same rate as the monitor refresh and stimulus update rate.

The translation of the observer's head produced a relative rotation of the simulated planar surface of about 3.32 about the vertical axis, regardless of surface slant. The maximum lateral head shift was equal to 50 mm. In the passive-vision condition, the optic flows were generated by replaying the 2D transformations generated by the corresponding active-vision trials. The horizontal translation component of the optic flow was removed by assuming that the cyclopean line of sight of the active observers was always aligned with the centre on the planar surface, regardless of actual head position and surface slant [37].

The physical planar surface was painted black and randomly covered with phosphorescent dots. With respect to the virtual surface, the physical surface was covered by larger dots (about 5 mm) having an irregular shape, a lower density (about 13 dots were visible through the irregular aperture), and providing texture cues (*i.e.*, dot foreshortening) consistent with a slanted 3D planar surface. Given the smaller viewing distance (760 mm), the constant amount of lateral head shift produced a relative rotation of the surface about the vertical axis of 3.76.

During the experiment, the room was completely dark. Peak head translation velocity was either 285.6 mm/s or 57.7 mm/s. Depending on the head translation velocity, on each trial the stimulus was visible for about 3.0 s or 11.1 s.

### Design

Each observer participated in three experimental blocks in the following order: Active-Vision with a Virtual surface (AVV), Passive-Vision with a Virtual surface (PVV), and Active-Vision with a Physical surface (AVP). Participants were randomly assigned either to the “normal” or to the “slow” head translation velocity conditions. Each AVV and PVV block comprised 25 trials (5 repetitions of 5 simulated slants magnitudes). The AVP block comprised 16 trials (4 repetitions of 4 slant magnitudes). In the PVV block, the stimuli generated in the AVV block were shown again in random order. The completion of each block of trials required about 30 minutes.

### Procedure

**Figure 10:**
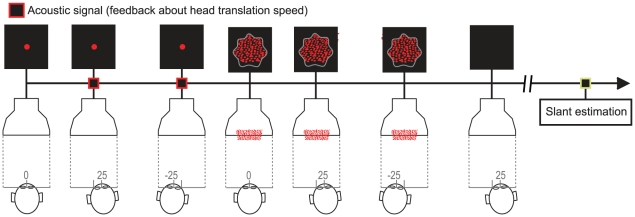
Temporal structure of the sequence in each trial. Schematic representation of one trial of our experiment. Here we consider the case of the acting viewing of a virtual surface.

Participants were tested individually in total darkness, so that only the stimulus displays shown on the CRT screen, or the luminous dots on the physical surface, were visible. In the AVV and AVP blocks, observers viewed the stimuli while making back-forth lateral head translations. The observer's head was supported by an horizontally extended chin-rest allowing lateral movements of 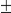 60 mm. An acoustic feedback signaled whether the average head shift velocity exceeded the range of 83 mm/s 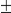 40 mm/s (“normal” speed) or 20 mm/s 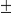 10 mm/s (“slow” speed). The stimulus display appeared on the screen when participants completed 2 consecutive back-and-forth translations at the required velocity and disappeared after 5.5 back-and-forth translations. After the stimulus disappeared, participants stopped moving their head and provided a verbal judgment of the amount of perceived surface slant (0° indicating a frontal-parallel surface, 90° indicating a surface parallel to the 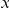,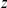 plane) – see Figure 10. In the PVV condition, participants were required to remain still for the entire duration of each trial.

Each experimental session was preceded by a preparatory session in which the participant's inter-pupillary distance was measured, the instructions were provided, and training about the appropriate head translation velocity and the magnitude estimation task was provided. Participants were trained in the magnitude estimation task by completing two blocks of 20 trials each. In one block, they were required to generate an angle between two segments on a computer screen after being prompted by a random number in the range 0–360. In the other block, they were required to estimate a random angle depicted to the screen. The relationship between the response and the test values was analyzed with a linear regression. Only participants who met performance criteria of a slope in the interval [0.9, 1.1] and an intercept in the interval [−0.3, 0.3] entered the experimental session.

**Figure 11:**
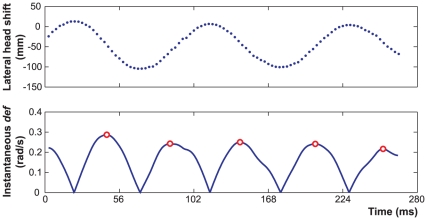
Example of instantaneous *def* as a function of time. The maximum of this temporal profile (averaged across the *def* cycles) was computed for each trial of the experiment and was used to test the Bayesian model of Eq. 15.

The maximum value of *def* was extracted in each trial from the instantaneous profile of the deformation component of the optic flow by following the procedure illustrated in Figure 11. These *def* values were then used to test the prediction of Eq. 15.

### Statistical Analyses

Statistical analyses were performed by means of Linear Mixed-Effects models with participants as random effects and 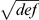, simulated slant, and head translation velocity (“normal”, “slow”) as fixed effects. We evaluate significance by computing the deviance statistic (minus 2 times the log-likelihood; change in deviance is distributed as chi-square, with degrees of freedom equal to the number of parameters deleted from the model) and with the help of 10,000 samples from the posterior distributions of the coefficients using Markov chain Monte Carlo sampling. From these samples, we obtained the 95% Highest Posterior Density confidence intervals, and the corresponding two-tailed 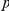-values. Several indexes have been proposed to measure the prediction power and the goodness-of-fit for linear mixed models (*e.g.*, Sun, Zhu, Kramer, Yang, Song, Piepho, & Yu, 2010). Here, we measure the goodness of fit as 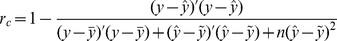, where 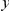 is an 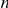 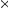 1 vector, 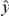 are the fitted values, 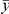 is the mean of 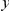, and 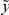 is the mean of 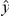 (Vonesh, Chinchilli, & Pu, 1996). The 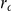 statistic can be interpreted as a measure of the degree of agreement between the observed values and the predicted values. The possible values of 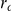 lie in the range 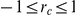.
